# Supplementary material for: The causal relationship between the human gut microbiota and pyogenic arthritis: a Mendelian randomization study
Source: Front Cell Infect Microbiol. 2024 Nov 26;14:1452480. doi: 10.3389/fcimb.2024.1452480 (PMC11629706; doi:10.3389/fcimb.2024.1452480)
Supplement: Supplementary file 1 [file DataSheet1.zip › mendelian test/ebi-a-GCST90027504.csv_forest.pdf]

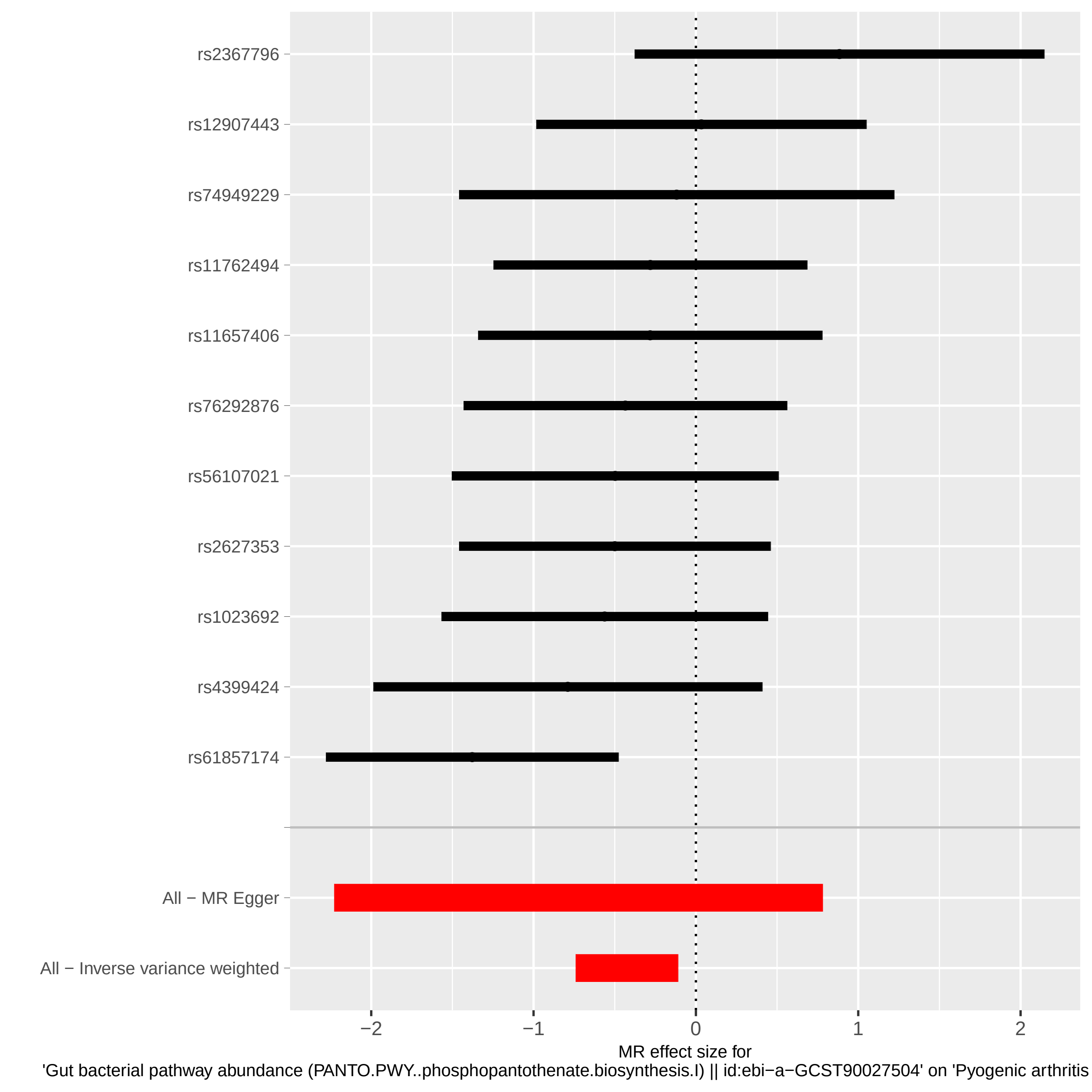
rs2367796
rs12907443
rs74949229
rs11762494
rs11657406
rs76292876
rs56107021
rs2627353
rs1023692
rs4399424
rs61857174
All − MR Egger
All − Inverse variance weighted
−2
−1
0
1
2
MR effect size for
'Gut bacterial pathway abundance (PANTO.PWY..phosphopantothenate.biosynthesis.I) || id:ebi−a−GCST90027504' on 'Pyogenic arthritis
